# Industrial Sustainable Decrystallizing Formulation to Enhance Dissolution of Candesartan Cilexetil: Overcoming Limitations of Traditional Solid Dispersion Approaches

**DOI:** 10.3390/pharmaceutics17091214

**Published:** 2025-09-17

**Authors:** Mohamed A. Ibrahim, Abdelrahman Y. Sherif, Doaa Hasan Alshora

**Affiliations:** Department of Pharmaceutics, College of Pharmacy, King Saud University, Riyadh 11451, Saudi Arabia; dalahora@ksu.edu.sa

**Keywords:** decrystallizing formulation, solid dispersion, candesartan cilexetil, industrial manufacturing score

## Abstract

**Background/Objectives**: Conventional solid dispersion methods face significant industrial limitations, including thermal degradation, residual organic solvents, and complex preparation processes. This study presents a novel decrystallizing formulation using poloxamer and propylene glycol that remains solid during storage but liquefies at physiological temperature (37 °C). **Methods**: Decrystallizing formulations containing various poloxamer types (407 and 188) at different concentrations (5–25% w/w) were prepared and assessed for decrystallization temperature, decrystallization time, and drug solubility. The optimal formulation was further characterized using FTIR analysis, as well as in vitro liquefaction performance and dissolution studies. Finally, the industrial sustainability of the decrystallizing formulation was assessed against conventional methods. **Results**: Poloxamer 407 exhibited higher decrystallization temperature, longer decrystallization time, and superior solubilization capacity compared to Poloxamer 188. Maximum drug solubility (5.51 ± 0.08 mg/g) was achieved at 20% w/w of poloxamer 407 with a decrystallization temperature of 37 °C, and it took 216 s for decrystallization. FTIR spectroscopy confirmed hydrogen bonding interactions, which are responsible for temperature-dependent phase transitions. The decrystallizing formulation showed remarkable improvement in dissolution efficiency (80.6 ± 3.9%) compared to the raw drug (1.8 ± 0.8%), a physical mixture (11.1 ± 6.0%), and a marketed tablet (30.8 ± 2.2%). **Conclusions**: The current decrystallizing formulation offers a promising approach for improving the bioavailability of poorly water-soluble drugs and tackling the limitations of conventional methods. Moreover, it provides additional advantages in terms of industrial sustainability for continuous production compared to conventional approaches.

## 1. Introduction

The reported diminished bioavailability of hydrophobic drug molecules is usually ascribed to their low solubility within the hydrophilic microenvironment of the gastrointestinal tract [1]. This challenge hinders the utilization of hydrophobic drugs while treating various medical conditions and limits their application in pharmaceutical development [2]. Therefore, numerous approaches have been utilized to overcome the aforementioned limitation of poorly water-soluble molecules [3,4]. Among them, the solid dispersion approach has emerged to enhance the dissolution and bioavailability of poorly water-soluble drugs [5].

Solid dispersion is usually composed of drug molecules that are molecularly dispersed within the matrix of hydrophilic polymers [6,7]. This reduction in the drug’s particle size to the molecular level increases the surface area available for dissolution [8]. Furthermore, this could facilitate the transformation of the drug from a crystalline to an amorphous state, which improves drug dissolution [9]. Additionally, the presence of hydrophilic polymers improved the wettability of the incorporated drug owing to its hygroscopic nature [10]. Moreover, the hydrophilic polymers could have surfactant-like activity, maintaining the drug’s solubilized state within the gastrointestinal tract and preventing precipitation [11]. 

Various methods are used to prepare solid dispersion, such as microwave, lyophilization, spray drying, and hot-melt extrusion [12]. However, these conventional methods face several limitations that can impact their industrial application. Potential thermal degradation and incomplete drug amorphization within the prepared solid dispersion are major drawbacks of the microwave method [13]. On the other hand, time consumption, high production cost, and low production yield limit the application of lyophilization in pharmaceutical production [14]. In addition, the presence of residual organic solvents in the prepared formulation using spray drying methods raises concerns about their usage during the preparation of the solid dispersion [15]. Regarding hot-melt extrusion, exposure of drugs to higher temperatures may result in the degradation of thermosensitive drugs [16].

These limitations underscore the need for alternative approaches to address the aforementioned challenges of the traditional preparation method. In this context, a novel decrystallizing formulation composed of a thermoresponsive polymer (poloxamer) and a cross-linking agent (propylene glycol) has been proposed to overcome the limitations of conventional approaches. This formulation exists in a solid state at room temperature and can be converted to a liquid state upon exposure to body temperature. The proposed approach offers additional benefits, including a simple preparation method that avoids the use of energy-consuming instruments and the use of organic solvents, which have a harmful impact on the environment. 

Candesartan cilexetil, an antihypertensive therapeutic agent, decreases blood pressure through an antagonistic influence on the angiotensin II receptor [17]. The reported potent effect of candesartan cilexetil resulted in various desirable outcomes in clinical settings [18]. It has been reported that candesartan cilexetil has a cardioprotective effect, reducing the incidence of heart failure [19]. A clinical study revealed that it provides a desirable nephroprotection against renal injury and neuroprotection effects [20,21]. 

However, the low aqueous solubility of candesartan cilexetil (0.0595 mg/mL) necessitates the demand for amorphization technology to overcome this issue [22]. This negatively impacts oral bioavailability, which is reported to be 40% of the administered dose [23]. Therefore, various approaches, such as solid dispersion [24], complexation [25], and lipid-based formulation [26], have been employed to enhance dissolution. However, demand persists for an alternative approach to address the industrial limitations of organic solvent residues, thermal degradation risks, and complex manufacturing processes. Therefore, the selection of candesartan cilexetil in the present study makes it an ideal model to resolve limitations of conventional approaches. 

The current study aims to develop a proposed novel formulation containing candesartan cilexetil to investigate its ability to enhance the dissolution of poorly water-soluble drug molecules. To achieve this, various types of poloxamer were used at various concentrations to prepare formulations. Moreover, the prepared formulation was subjected to pharmaceutical characterization in terms of decrystallizing temperature, decrystallizing time, and solubility to select the optimum formulation. Finally, the in vitro liquification and dissolution performance of the optimized formulation were tested to study the drug dissolution behavior from the proposed formulation. 

## 2. Materials and Methods

### 2.1. Materials

Riyadh Pharma (Riyadh, Saudi Arabia) generously supplied candesartan cilexetil. Propylene glycol was obtained from Winlab Laboratory (Leicestershire, UK). Sigma-Aldrich (St. Louis, MO, USA) provided both ammonium formate and formic acid, which were used for the preparation of the aqueous component of the mobile phase. AppliChem Panreac (Darmstadt, Germany) provided acetonitrile, which was used as an organic component of the mobile phase. 

### 2.2. UPLC Method for Drug Quantification

The developed ultra-performance liquid chromatography (UPLC) was implemented using a Dionex UltiMate 3000 system (Thermo Scientific, Bedford, MA, USA) to determine the candesartan cilexetil concentration within the analyzed samples during solubility and dissolution studies. The mobile phase (10 mM ammonium formate buffer: 0.1% formic acid: acetonitrile; 36: 4: 60% v/v) was eluted using a Dionex UltiMate 3000 system (Thermo Scientific, Bedford, MA, USA) at a 0.4 mL/min flow rate through a connected HSS T3 1.8 µm (2.1 × 50 mm) column. The temperature of the utilized column was preheated to 25 °C during analysis of the injected samples. A connected Dionex Photodiode Array (PDA) detector (Thermo Scientific, Bedford, MA, USA) was set at 254 nm to measure the absorbance of candesartan cilexetil within samples. The UPLC method and data analysis were performed using Chromeleon software version 5.

### 2.3. Selection of Cross-Linking Agent

This study aimed to select an appropriate cross-linking agent and identify the agent that facilitates the solidification of the prepared formulation. Herein, three potential cross-linking agents (isopropanol, propylene glycol, and glycerol) were screened based on their expected hydrogen bonding formation capability with poloxamer. A 10% w/w poloxamer 407 was dissolved in the cross-linking agent individually to select the optimum one. The solubility of poloxamer 407 was tested at two temperatures (40 °C and 8 °C) using an incubator and a refrigerator, respectively. These two temperatures assess the solubilization power of the cross-linking agent and the crystallization behavior of the prepared mixture. These steps were repeated three times to ensure the final appearance of the mixtures when exposed to multiple cycles. 

### 2.4. Preparation of Decrystallization Formulation

Decrystallization formulations were prepared to assess the impact of polymer concentration on their pharmaceutical behavior. For each polymer (Poloxamer 407 and 188), five decrystallization formulations were prepared at concentrations of 5, 10, 15, 20, and 25% w/w following poloxamer dissolution in propylene glycol, as detailed in Table 1. All ten formulations were evaluated for decrystallization temperature, decrystallization time, and drug solubility to select the optimal formulation composition.

### 2.5. Decrystallization Temperature 

The decrystallization temperature for the prepared formulations was measured to determine the temperature at which the decrystallizing formulation liquefies in vivo. Within a glass test tube, approximately one gram of the decrystallizing formulation was placed and stored in the refrigerator to solidify. The temperature of the decrystallizing formulation was controlled by placing a rack containing test tubes in a water bath set at 28 ± 0.1 °C. After a 5-min equilibration period, a visual assessment of the formulations’ appearance was performed to determine if they had decrystallized. Afterward, the temperature of the water was increased by 0.5 ± 0.1 °C after each assessment until the decrystallization temperature for all formulations was determined. 

### 2.6. Decrystallization Time 

For the assessment of decrystallization time under in vivo conditions, water temperature was maintained at 37 ± 0.1 °C. The time required for the prepared formulation to decrystallize was evaluated separately. Test tubes were immersed in the water bath to a depth ensuring complete submersion of the formulation sample within a controlled temperature. Continuous visual monitoring for the immersed formulation was performed. The endpoint was determined once the formulation’s physical appearance changed to a liquefied state.

### 2.7. Solubility 

The solubility of candesartan cilexetil within the decrystallizing formulation was assessed using a simple mixing method. An excess amount of candesartan cilexetil was mixed with the decrystallizing formulation at a stirring rate of 1000 rpm for 24 h. To precipitate the undissolved drug, the mixture was centrifuged the next day for 10 min at 10,000 rpm. Drug solubility was assessed by determining drug concentration in the supernatant using the UPLC method after proper dilution.

### 2.8. FTIR

A PerkinElmer Spectrum-100 Spectrometer (Waltham, MA, USA) was used to study the chemical interaction between poloxamer and propylene glycol. Fourier transform infrared (FTIR) spectra of poloxamer, propylene glycol, their physical mixture, solid decrystallizing formulation, and liquid decrystallizing formulation were scanned in the wavenumber range of 800–4000 cm^−1^. Each sample was measured in duplicate. Samples were placed directly onto the diamond ATR crystal, and gentle pressure was applied using the sampling accessory to ensure good contact between the sample and crystal surface. Data acquisition and processing were performed using Spectrum PerkinElmer, Inc. software version 6.3.5. 

### 2.9. In Vitro Liquefication Performance

This experiment was conducted on the decrystallizing formulation to guarantee a complete liquification behavior that occurs following oral administration under physiological conditions. The temperature of the dissolution vessel containing 200 mL of phosphate buffer was preheated to 37 ± 0.5 °C. A hard gelatin capsule filled with the decrystallizing formulation surrounded by a cylindrical sinker was immersed in the dissolution vessel. Throughout the liquification process, images were captured for photographic documentation of physical appearance changes in the formulation.

### 2.10. Dissolution Study 

The current study investigates the impact of decrystallizing formulation on the dissolution performance of candesartan cilexetil. The dissolution study was carried out using a Type II apparatus assembled paddle (LOGAN Inst. Corp., Franklin, NJ, USA). The dissolution medium consisted of 900 mL of phosphate-buffer solution (pH 6.8), which was prewarmed to 37.0 ± 0.5 °C prior to the experiment. Hard gelatin capsules containing either 8 mg of pure candesartan cilexetil or an equivalent amount of the drug in a decrystallized formulation were used. The dissolution test was performed under paddle rotation at 50 rpm. Sample collections were taken at specified time points (5, 15, 30, 45, and 60 min), and drug concentration was determined using the developed UPLC analytical method. The tolerance level for successful formulation was considered to be not less than 80% of the drug dissolved within 45 min. 

Various dissolution parameters were calculated to investigate the impact of formulation type on the dissolution of candesartan cilexetil. The initial dissolution rate was calculated using equation (1) over the first 5 min to provide a clear impression of the formulation’s efficiency in enhancing drug dissolution. However, the relative dissolution rate was calculated using equation (2) over the entire 60 min. This is attributed to the negligible drug dissolution from raw candesartan cilexetil occurring within the initial 15 min, which prevents the calculation of dissolution rate.

Initial dissolution rate (IDR):

(1) $$IDR=\frac{Q_{t}}{t}$$

Here,

IDR = Initial dissolution rate (%/min);

$Q_{t}$ = Percentage of drug released in a specific time (%);

t = Time period (min).

Relative dissolution rate (RDR):

RDR = DR test/DR reference(2)

Here,

DR test = Dissolution rate of test formulation (%);

DR reference = Dissolution rate of raw drug (%).

### 2.11. Industrial Manufacturing Score Assessment 

The industrial sustainability of the decrystallizing formulation was evaluated based on comparing five critical parameters across conventional solid dispersion techniques: organic solvent usage, energy consumption, equipment complexity, processing time, and waste generation. Each parameter was scored on a scale of 1–5 (1 = least sustainable, 5 = most sustainable) based on established criteria as shown in Table 2. The total sustainability score was calculated as the sum of individual parameter scores. 

## 3. Results and Discussion

### 3.1. UPLC Method for Drug Quantification

**Figure 1:**
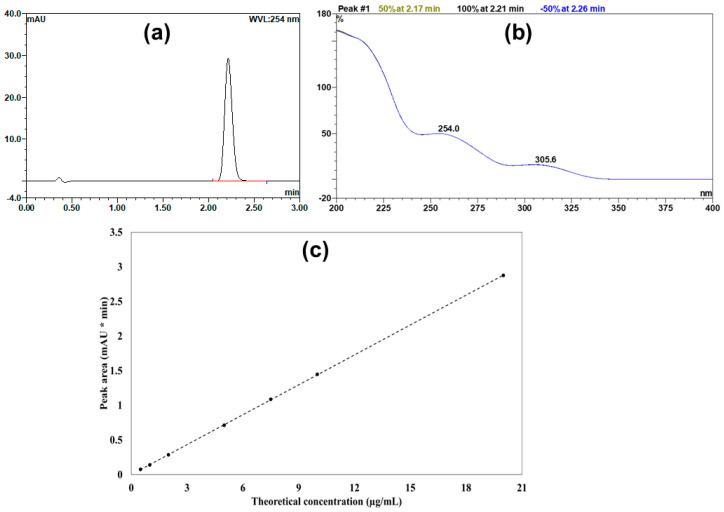
(**a**) Representative UPLC chromatogram showing the candesartan cilexetil peak. (**b**) The UV absorption spectrum of the candesartan cilexetil. (**c**) The calibration curve shows theoretical drug concentration against the measured peak area.

The developed UPLC method produced a well-defined peak of candesartan cilexetil with a retention time of 2.2 min (Figure 1a). Moreover, Figure 1b displays the UV absorption spectrum of the obtained peak, highlighting the maximum characteristic λ max of candesartan cilexetil at 254.0 nm. In addition, the constructed calibration curve exhibited remarkable linearity, achieving a regression coefficient (r^2^) value of 0.9999 over the selected range (0.5–20 μg/mL). Figure 1c presents the contracted calibration curve, which was used to quantify candesartan cilexetil within samples. Additionally, Table 3 summarizes analytical parameters for the developed UPLC method. 

### 3.2. Selection of Cross-Linking Agent

**Figure 2:**
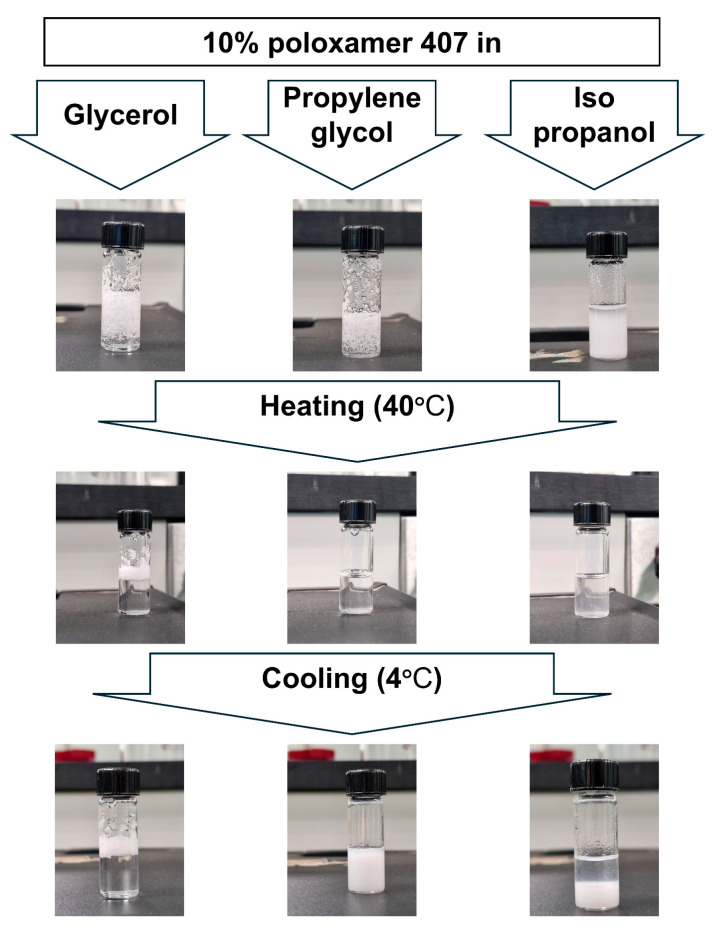
Physical appearance of 10% w/w poloxamer 407 in glycerol, propylene glycol, and isopropanol after mixing (top), at 40 °C (middle), and at 4 °C (bottom).

The present test was performed to select the appropriate cross-linking agent for the thermoresponsive polymer used. Three cross-linking agents (isopropanol, propylene glycol, and glycerol) were used to study their impact on the thermoresponsive behaviors of poloxamer polymers. For screening purposes, poloxamer 407 was dissolved in the selected cross-linking agents at a concentration of 10% w/w. The current study revealed a significant difference in the solubilization behavior of poloxamer 407 in selected solvents, as demonstrated in Figure 2. The physical appearance showed that poloxamer 407 dissolved in both isopropanol and propylene glycol upon heating to 40 °C, while glycerol failed to dissolve the polymer at both low and high temperatures.

**Figure 3:**
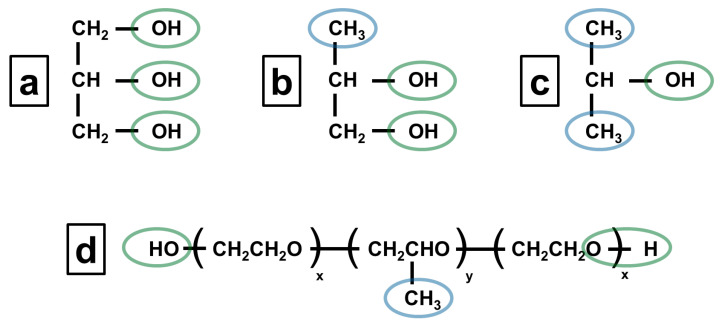
Chemical structure of (**a**) glycerol, (**b**) propylene glycol, (**c**) isopropanol, and (**d**) poloxamer.

The scientific explanation for these observations can be understood from the structural analysis presented in Figure 3. Poloxamer 407 dissolutions in isopropanol and propylene glycol are facilitated by the presence of both hydrophilic hydroxyl groups (OH) and hydrophobic methyl groups (CH_3_), indicated by green and blue circles, respectively. The hydrophilic portions of both solvents could interact favorably with the polyethylene oxide segments of poloxamer, while the hydrophobic regions interact with the polypropylene oxide blocks. On the other hand, the absence of a hydrophobic part in glycerol prevents the effective solubilization of poloxamer.

Upon heating, both isopropanol and propylene glycol dissolve poloxamer 407 through favorable molecular interactions. At elevated temperatures, poloxamer chains adopt micellar structures with hydrophobic polypropylene oxide cores surrounded by hydrophilic polyethylene oxide shells [27,28]. This allows dissolution of poloxamer in both solvents through miscibility between the hydrophilic hydroxyl groups of both agents. 

**Figure 4:**
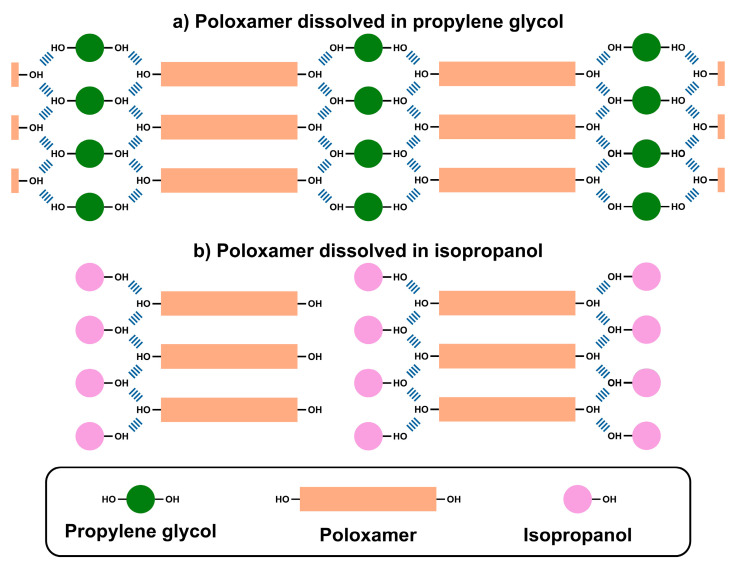
Molecular interactions of poloxamer with (**a**) propylene glycol and (**b**) isopropanol.

However, the cooling process drives the conversion of polymer assembly from micellar to monomeric units [29,30]. Therefore, poloxamer and propylene glycol form a highly cross-linked network (Figure 4a) through hydrogen bonding between the hydroxyl groups of propylene glycol and the terminal hydroxyl groups of poloxamer. Consequently, this mixture forms a three-dimensional crystalline structure as shown in Figure 2. In contrast, Figure 4b shows that isopropanol cannot form the same three-dimensional network due to the presence of one hydroxyl group within its structure. Therefore, the isopropanol-poloxamer system precipitates at the bottom upon cooling and cannot form a similar crystalline structure in a system containing propylene glycol. In conclusion, propylene glycol was chosen as the optimum cross-linking agent for our proposed decrystallizing system owing to its capability to remain solid when stored at low temperature, while it converts to a liquid state upon exposure to high temperature. 

### 3.3. Decrystallization Temperature 

**Figure 5:**
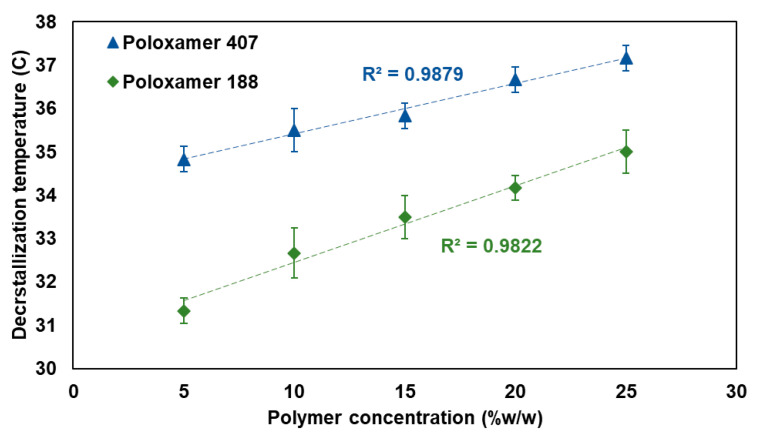
The relationship between polymer concentration and decrystallization temperature for poloxamer 407 (blue) and poloxamer 188 (green).

The decrystallization temperature was performed to determine the temperature at which the decrystallizing formulation switches its physical state from solid to liquid. Figure 5 presents the relationship between polymer concentration and the decrystallization temperature for both poloxamer 407 and poloxamer 188-based formulations. The results demonstrated a clear concentration-dependent increment in decrystallization temperature for both types of polymers used. For poloxamer 407, the solid-to-liquid transition temperature ranged from 35.17 ± 0.29 °C at a 5% w/w concentration to 37.33 ± 0.29 °C at a 25% w/w concentration, exhibiting an excellent linear correlation (R^2^ = 0.9660). Similarly, poloxamer 188 exhibited transition temperatures ranging from 31.33 ± 0.29 °C at a 5% w/w concentration to 35.00 ± 0.50 °C at a 25% w/w concentration, with a good linear relationship (R^2^ = 0.9822). 

The present finding reflects the enhanced hydrogen bonding interaction between the used components and increased cross-linking degree at higher concentrations. This could be ascribed to the reported positive influence of increased polymer concentration on the developed intermolecular cross-linking interactions [31,32]. In addition, Poloxamer 407-based formulation consistently required higher temperatures for the liquid phase transition compared to its counterpart, a decrystallizing formulation containing Poloxamer 188 at the same concentration level. This increment in measured decrystallizing temperature can be attributed to the higher molecular weight of poloxamer 407, which forms a strong cross-linking matrix. This agrees with previously reported studies, which showed that increasing the molecular weight of the polymer builds stronger intermolecular interactions and requires additional thermal energy to disrupt them. The measured data for the decrystallizing temperature agree with the reported positive effect of polymeric molecular weight on intermolecular interactions [33]. 

### 3.4. Decrystallization Time 

**Figure 6:**
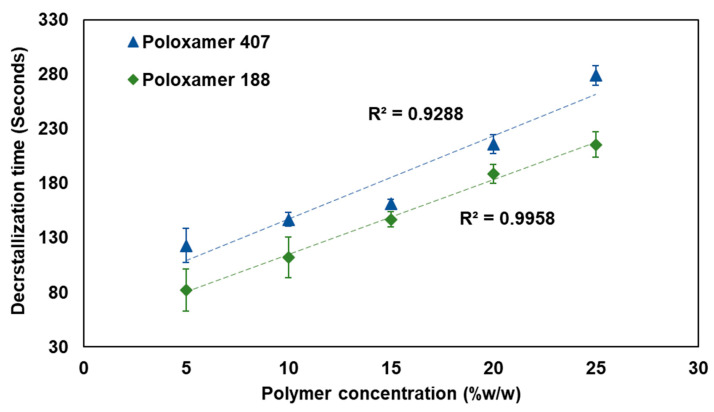
The relationship between polymer concentration and decrystallization time for poloxamer 407 (blue) and poloxamer 188 (green).

The decrystallization time was conducted to determine the duration required for the complete phase transition of the decrystallizing formulation from a solid to a liquid state at physiological conditions. Figure 6 illustrates the relationship between polymer concentration and decrystallization time for both poloxamer 407 and poloxamer 188-based formulations. The data obtained showed a direct concentration-dependent increase in decrystallization time for both polymer types. For poloxamer 407, linear correlation (R^2^ = 0.9288) was observed in the measured decrystallization time, ranging from 123 ± 15.59 to 279 ± 9.16 s, when polymer concentration increased from 5 to 25% w/w, respectively. Similarly, poloxamer 188-based formulations exhibited strong linearity (R^2^ = 0.9958) in measured decrystallization times, ranging from 82 ± 19.47 to 215.67 ± 11.59 s, as the polymer concentration increased from 5 to 25% w/w, respectively. 

The observed concentration-dependent increase in decrystallization time reflects the formation of strong intermolecular bonds at higher polymer concentrations. Therefore, it requires a longer duration of period to entirely disrupt the formed intermolecular bonds and convert to a liquid phase. Poloxamer 407-based formulations dependably require longer decrystallization times compared to poloxamer 188-based formulations at equivalent concentrations. This difference can be attributed to the ability of the higher molecular weight polymer (Poloxamer 407) to form stronger intermolecular bonds compared with its counterpart, the polymer with a lower molecular weight (Poloxamer 188). 

### 3.5. Solubility 

**Figure 7:**
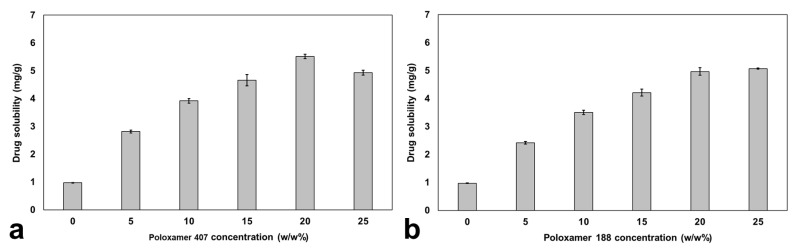
Solubility of candesartan cilexetil in propylene glycol containing (**a**) poloxamer 407 and (**b**) poloxamer 188 at various concentrations.

Figure 7 shows the solubility of candesartan cilexetil in propylene glycol and the decrystallizing formulation containing poloxamer 407 and 188 at different concentrations. The solubility study in propylene glycol was crucial in establishing the baseline drug-carrying capacity before polymer addition. It was found that candesartan cilexetil exhibited low solubility in propylene glycol, with a value of 0.97 ± 0.01 mg/g. However, the solubilization of candesartan cilexetil in decrystallizing formulations containing both polymers positively increased candesartan cilexetil solubility, with a superior impact of Poloxamer 407 at the same polymer concentration. The enhanced solubilization capacity of poloxamer 407 can be attributed to its longer polypropylene oxide chains, which provide a more hydrophobic environment for the solubilization of candesartan cilexetil compared to poloxamer 188. Based on the triblock structure, the calculated ratio of propylene oxide: ethylene oxide is 1:3.5 for poloxamer 407 compared to 1:6 for poloxamer 188 [34]. This enables poloxamer 407 to form larger micellar structures with a hydrophobic core where lipophilic candesartan cilexetil molecules can be effectively solubilized. 

### 3.6. Selection of Optimized Formulation

The selection criteria were performed based on a systematic evaluation of all measured responses (decrystallization temperature, decrystallization time, and solubility). Using a formulation with a high decrystallization temperature could prevent premature conversion to the liquid state before administration. Moreover, the formulation exhibits a decrystallization temperature at body temperature, ensuring conversion to the liquid state in vivo. Furthermore, formulation with low decrystallization time guarantees rapid conversion to the liquid state in vivo after administration. However, all formulations decrystallize within less than 5 min. Hence, this parameter was excluded from the selection criteria. Finally, formulations with high drug solubilization capacity reduce the total dosage volume and enhance pharmaceutical applicability. Consequently, the formulation containing 20% w/w of poloxamer 407 was selected as the optimized one because it achieved the highest drug solubilization capacity, with a value of 5.51 ± 0.08 mg/g, and exhibited a decrystallization temperature of 37 °C. Moreover, the selected decrystallizing formulation exhibited complete liquification within 5 min. 

### 3.7. FTIR

**Figure 8:**
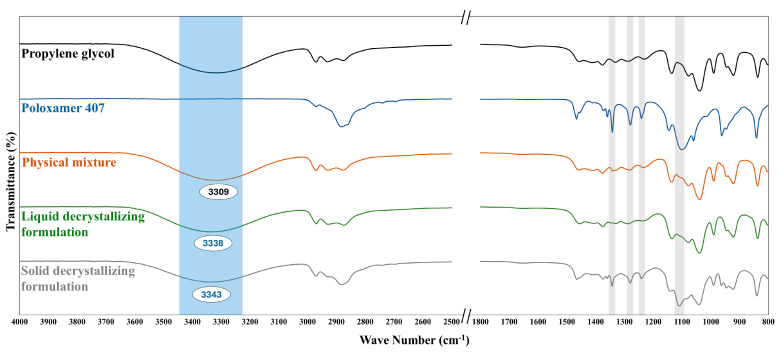
FTIR spectrum of propylene glycol, Poloxamer 407, their physical mixture, liquid decrystallizing formulation, and solid decrystallizing formulation.

FTIR spectroscopy was used to elucidate the expected molecular interactions and structural organization of poloxamer 407 within propylene glycol that triggered the thermal behavior of the prepared decrystallizing formulation. Figure 8 shows the FTIR spectra of propylene glycol, Poloxamer 407, their physical mixture, liquid decrystallizing formulation, and solid decrystallizing formulation. The propylene glycol spectrum exhibited characteristic absorption peaks, including a broad peak at 3319 cm^−1^ (O-H stretching) and sharp peaks at 2970–2876 cm^−1^ (-CH_3_ and -CH_2_ stretching vibrations). Moreover, the fingerprint region showed distinctive vibrations at 1231 cm^−1^ (C-O stretching of secondary alcohols) and 1136 cm^−1^ (C-O stretching of primary alcohols) [35,36]. The spectrum of Poloxamer 407 showed characteristic peaks at 2881 cm^−1^ (C-H symmetric stretching) and peaks at 1466 cm^−1^ (CH_2_ bending vibrations). Moreover, the fingerprint region displayed distinctive bands at 1342 cm^−1^ and 1279 cm^−1^ (CH_2_ wagging and twisting vibrations), and 1100 cm^−1^ (C-O-C stretching). Additionally, characteristic vibrations were observed at 1241 cm^−1^ (C-O stretching), representing the ether and terminal hydroxyl functionalities of the polymer backbone [30,37]. The physical mixture spectrum displays the characteristic peaks of propylene glycol and poloxamer 407, with minimal intensity of the latter. This could be attributed to the dilution effect produced by the former and demonstrates the absence of chemical interaction between the two agents.

FTIR spectrum of liquid decrystallizing formulation containing poloxamer 407 dissolved within propylene glycol showed characteristic changes compared to the physical mixture. Blue shift of the O-H stretching peak from 3309 to 3338 cm^−1^ (indicated by blue box) was observed with no significant change in other peaks compared to the physical mixture. This finding can be attributed to the disruption of propylene glycol self-association and the formation of new bonds with poloxamer 407, which is present in micellar structures. This shifting could be attributed to the formation of the H-bond between the hydroxyl groups of propylene glycol and poloxamer. In the liquid formulation, the intensities of CH_2_ wagging (1342 cm^−1^), CH_2_ twisting (1279 cm^−1^), and C-O-C stretching (1100 cm^−1^) were reduced, indicating the conversion of poloxamer to a micelle structure.

The FTIR spectrum of the solid decrystallizing formulation was consistent with the previously proposed mechanism of transition from a micellar to a monomeric state upon cooling. The results showed a blue shift of the O-H stretching peak, similar to that of the liquid decrystallizing formulation, from 3309 to 3343 cm^−1^ (indicated by blue box). This indicated the presence of H-bonds between monomeric polymer units and propylene oxide, as illustrated in Figure 4. Furthermore, the characteristic peaks of poloxamer 407, including CH_2_ wagging and twisting, as well as C-O-C stretching (indicated by gray boxes), were significantly enhanced in the solid decrystallizing formulation compared to the liquid formulation. The highlighted gray boxes indicated that the poloxamer peaks in the solid decrystallizing formulation were enhanced compared to those in the liquid decrystallizing formulation. This also confirms the change of poloxamer to a monomeric state where its characteristic functional groups could be detected.

### 3.8. In Vitro Liquefication Performance 

**Figure 9:**
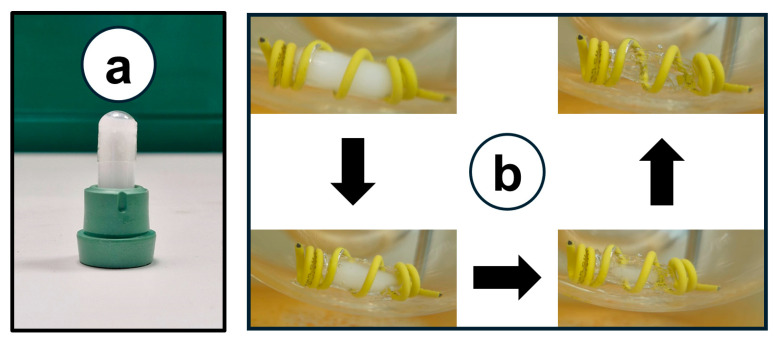
(**a**) Physical appearance of capsule-filled formulation. (**b**) Formulation melting once exposed to physiological body temperature (37 ± 0.5 °C).

Figure 9a shows the physical appearance of the solidified formulation, with no leakage risk, ensuring stability during storage. The capsule-filled formulation was surrounded by sinkers and placed in a 200 mL dissolution medium (pH 6.8) preheated to 37 ± 0.5 °C. Figure 9b presents the progression of formulation melting (indicated by arrows) when exposed to physiological body temperature. The current study confirmed that the decrystallizing formulation can liquify in vivo following oral administration.

### 3.9. Dissolution Study

**Figure 10:**
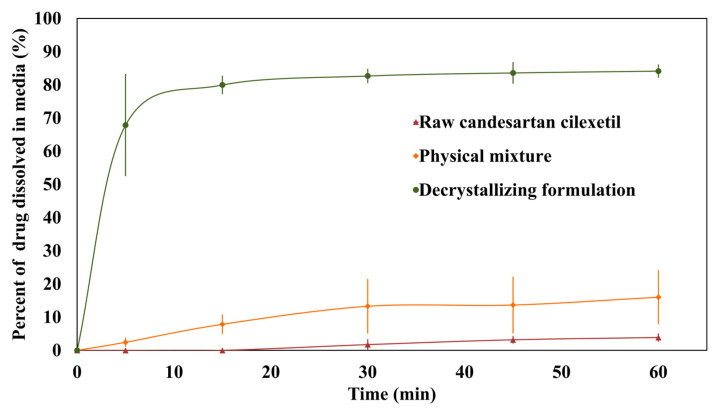
In vitro dissolution profile of raw candesartan cilexetil, physical mixture, and decrystallizing formulation.

Figure 10 shows the in vitro dissolution profiles of raw candesartan cilexetil, the physical mixture, and the decrystallizing formulation. Furthermore, Table 4 shows the calculated dissolution efficiency, initial dissolution rate, and relative dissolution rate for the tested agents. The results showed that 3.9 ± 1.1% of raw candesartan cilexetil was dissolved at the end of the study with a dissolution efficiency value of 1.8 ± 0.8%. The physical mixture and decrystallizing formulation significantly increased the dissolution efficiency of candesartan cilexetil to 11.1 ± 6.0 and 80.6 ± 3.9%, respectively. The results showed that the decrystallizing formulation was able to dissolve 83.6% of candesartan cilexetil within 45 min, which passed the tolerance level for successful formulation. Moreover, the initial dissolution rate analysis revealed substantial differences between formulations. The decrystallizing formulation exhibited the highest initial dissolution rate (13.58%/min), compared to negligible rates for the raw drug (0.00%/min) and physical mixture (0.48%/min). Additionally, the relative dissolution rate showed a 23.01-fold improvement for the decrystallized formulation compared to the raw drug.

The outstanding dissolution efficiency achieved by the decrystallizing formulation can be attributed to several mechanisms. Firstly, the prepared decrystallizing formulation exhibited an initial dissolution of candesartan cilexetil with a value of 67.9% within 5 min. This is clearly supported by the initial dissolution rate data, which showed an increase from 0.00%/min to 13.58%/min for the raw drug and the decrystallizing formulation, respectively. This indicates rapid liquefaction and immediate drug solubilization upon exposure to physiological temperature (37 °C), which agrees with the in vitro liquification performance study. Furthermore, complete formulation liquification overcomes the diffusional barriers present in the conventional matrix of conventional solid dispersion. The instant conversion from a solid to a liquid at body temperature provides immediate access to drug molecules dispersed in an amorphous state within the polymer matrix. In addition, the reported formation of a micellar structure by poloxamer chains creates a solubilization environment that significantly enhances drug dissolution. Moreover, a 23-fold increase in relative dissolution rate for the decrystallizing formulation compared with a 4.1-fold increase for the physical mixture suggests that candesartan cilexetil is solubilized within the matrix of the decrystallizing formulation. This provides indirect evidence regarding the molecular-level dispersion of the drug within the poloxamer matrix and its existence in an amorphous state. The results obtained showed that the prepared decrystallizing formulation is expected to improve the bioavailability of candesartan cilexetil significantly. This is consistent with a positive correlation between the enhanced bioavailability of drugs loaded within polymeric-containing delivery systems [38,39].

### 3.10. Comparative Assessment Against a Marketed Tablet 

**Figure 11:**
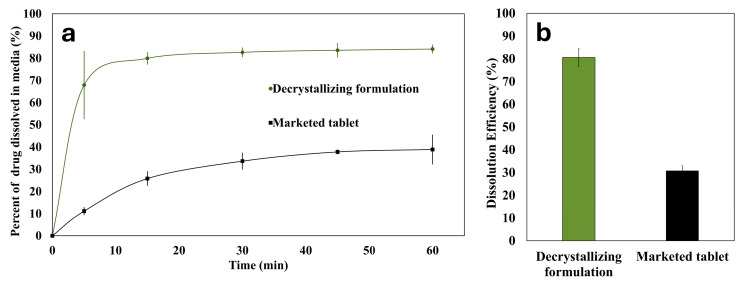
(**a**) In vitro dissolution profile and (**b**) dissolution efficiency of the decrystallizing formulation and the marketed tablet.

A comparative dissolution study for the prepared decrystallizing formulation against a commercial tablet is presented in Figure 11. The observed superior performance of the decrystallizing formulation highlights the limitations of conventional approaches that rely on disintegrants for tablets without the presence of precipitation inhibitors, such as poloxamer. This is expected to reduce inter-individual variability in bioavailability compared to conventional formulations. Moreover, using polymers with p-glycoprotein inhibitor activity, such as poloxamer, is expected to enhance the permeability and bioavailability of susceptible agents like candesartan cilexetil [40,41,42]. 

### 3.11. Industrial Manufacturing Score Assessment 

**Figure 12:**
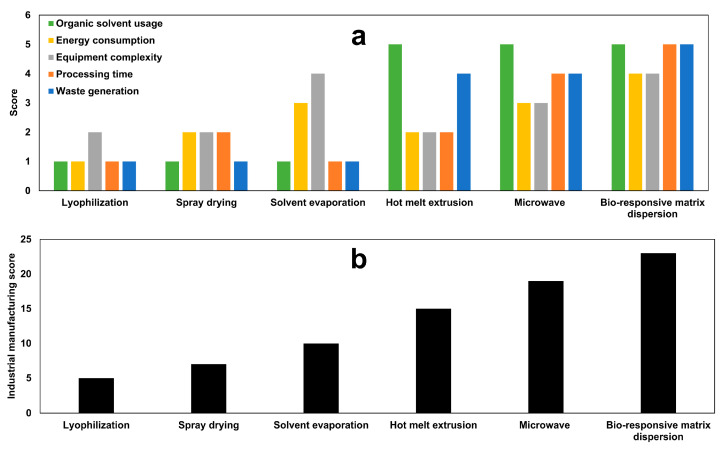
(**a**) Individual manufacturing and (**b**) industrial manufacturing scores for conventional solid dispersion technologies and decrystallizing formulation.

The current decrystallization formation was compared with the previously proposed methods in the literature. Figure 12a presents the scores for the individual assessment of the conventional solid dispersion technologies and decrystallizing formulation of five critical manufacturing parameters. Moreover, Figure 12b shows the overall industrial manufacturing score, which ranges from 5 to 23 points out of a maximum of 25 points. 

Lyophilization technology demonstrated poor manufacturing feasibility with the lowest industrial manufacturing score of 5 points. This is attributed to the use of organic solvents, which necessitate an energy-consuming sublimation process using an expensive instrument with a vacuum-controlled system [43,44]. In addition, the associated costs for collecting waste organic solvents during the sublimation process and disposal requirements increase the manufacturing expenses [45]. Moreover, the long processing times (24–72 h) further limit its applicability for mass production with low production cost [46]. 

Spray drying technology achieved a marginally improved performance with a score of 7 points, indicating a slight enhancement over lyophilization. This is ascribed to the continuous production capability and short processing time, which reduces the energy consumption during production [47]. However, it shares the limitations in terms of organic solvent usage, waste generation, and equipment complexity. Although the solvent evaporation method addresses the limitations of energy consumption and equipment complexity, it does not overcome other limitations [24,48]. Therefore, various approaches were invented to overcome these limitations.

Hot melt extrusion achieved a score of 15 owing to the lack of demand for organic solvents during the preparation of the solid dispersion, which reduces the generation of waste products during production [49,50]. Unfortunately, it did not achieve the desired improvement in terms of energy consumption, equipment complexity, and processing time. On the other hand, microwave technology tackles these limitations and achieves a score of 19. However, the final product for microwave is compact mass that requires further processing in terms of grinding and sieving to ensure uniformity of the final product [7,51]. This could slightly increase energy consumption, equipment complexity, and processing time during the production of a pharmaceutical dosage form. 

Decrystallizing formulation was invented to overcome the aforementioned limitation of conventional solid dispersion approaches. Eliminating the use of organic solvents during the preparation of solid dispersion reduces the generation of waste products during production. Moreover, rapid dissolution of poloxamer in propylene glycol with the production of a solution dosage form that is suitable for direct filling in a hand gelatin capsule. This avoids the powder processing requirements associated with other technologies, which reduces energy consumption through the use of complex equipment and minimizes processing time. Decrystallizing formulation offers substantial economic advantages through a noteworthy reduction in capital investment compared to conventional methods. 

## 4. Conclusions

In the present study, a novel decrystallizing formulation was successfully prepared to address the limitations of conventional solid dispersion. The optimized formulation, which consisted of 20% w/w poloxamer 407 dissolved in propylene glycol, exhibited a maximum drug solubility of 5.51 ± 0.08 mg/g. Moreover, the formulation exhibited decrystallization at physiological temperature (37 °C) within 5 min. FTIR analysis confirmed the presence of hydrogen bonding networks between poloxamer and propylene glycol, which are responsible for the temperature-dependent phase transitions. Furthermore, an in vitro dissolution study showed that the decrystallizing formulation had a dissolution efficiency (80.6 ± 3.9%) with a 45-fold improvement over raw candesartan cilexetil. The current decrystallizing formulation presents a promising approach for enhancing the bioavailability of poorly water-soluble drugs and addressing the limitations of conventional methods. It eliminates the use of organic solvents and avoids the need for high-energy processing or complex equipment. This represents a paradigm shift toward sustainable industrial pharmaceutical manufacturing processes which address the critical limitations of existing approaches. 

## Figures and Tables

**Table 1:** Composition of decrystallization formulations.

Poloxamer Type	Poloxamer Concentration (% w/w)	Propylene Glycol (% w/w)
Poloxamer 407	5	95
	10	90
	15	85
	20	80
	25	75
Poloxamer 188	5	95
	10	90
	15	85
	20	80
	25	75

**Table 2:** Scoring criteria for industrial sustainability assessment parameters.

Parameter	Score 5 (Most Sustainable)	Score 1 (Least Sustainable)
Organic Solvent Usage	Not required	Mandatory
Energy Consumption	Ambient temperature processing	Extreme heating/cooling systems
Equipment Complexity	Basic equipment	Highly specialized machinery
Processing Time	≤2 h	>12 h
Waste Generation	Minimal waste	Substantial hazardous waste

**Table 3:** Analytical parameters for the UPLC method for the quantification of candesartan cilexetil.

Parameter	Value
Linearity range (μg/mL)	0.5–20
regression coefficient	0.9999
Regression equation	y = 0.144x + 0.0004
Slope ± SD	0.144 ± 0.0004
Confidence interval of slope *	0.1429–0.1450
Standard error of the slope	0.00039
Intercept ± SD	−0.001 ± 0.0033
Confidence interval of intercept *	−0.0089–0.0098
Standard error of the intercept	0.0036
LLOD (μg/mL)	0.19 ± 0.03
LLOQ (μg/mL)	0.58 ± 0.08

* confidence interval 95%.

**Table 4:** Initial dissolution rate and relative dissolution rate of raw candesartan cilexetil, physical mixture, and decrystallizing formulation.

Tested Agent	Dissolution Efficiency (%)	Initial Dissolution Rate (%/min)	Relative Dissolution Rate
Raw candesartan cilexetil	1.8 ± 0.8	0.00 ± 0.0	1.00 ± 0.0
Physical mixture	11.1 ± 6.0	0.49 ± 0.2	4.13 ± 1.4
Decrystallizing formulation	80.6 ± 3.9	13.58 ± 3.0	23.01 ± 6.4

The initial dissolution rate was calculated within the first 5 min. The relative dissolution rate was determined over a 60-min period.

## Data Availability

The original contributions presented in this study are included in the article. Further inquiries can be directed to the corresponding authors.
